# Results of Chromosomal Microarray Need to Always Be Checked by (Molecular) Cytogenetics—Even If They Seem to Be Simple Deletions

**DOI:** 10.3390/genes16060714

**Published:** 2025-06-17

**Authors:** Thomas Liehr, Sylke Singer, Ulrike Mau-Holzmann, Stefanie Kankel, Niklas Padutsch, Luisa Person, Eva Daumiller, Uwe Kornak

**Affiliations:** 1 Jena University Hospital, Friedrich Schiller University, Institute of Human Genetics, Am Klinikum 1, 07747 Jena, Germany; stefanie.kankel@med.uni-jena.de (S.K.); niklas.padutsch@med.uni-jena.de (N.P.); luisa.person@med.uni-jena.de (L.P.); 2 Institut für Medizinische Genetik und Angewandte Genomik, Calwerstr. 7, 72076 Tübingen, Germany; sylke.singer@med.uni-tuebingen.de (S.S.); ulrike.mau@med.uni-tuebingen.de (U.M.-H.); 3 Genetikum Stuttgart, Lautenschlagerstr. 23, 70173 Stuttgart, Germany; daumiller@genetikum.de; 4 Universitätsmedizin Göttingen, Institut für Humangenetik, Heinrich-Düker-Weg 12, 37073 Göttingen, Germany; uwe.kornak@med.uni-goettingen.de

**Keywords:** chromosomal microarray, fluorescence in situ hybridization, loss of copy numbers, complex rearrangements, second test

## Abstract

**Background/Objectives:** Chromosome microarrays (CMAs) tend to be used as the first line test or as a test that does not require confirmation or verification by a second test. However, to understand the implications of a duplication or deletion for a family seeking genetic counseling, it is crucial to know the nature of the underlying chromosomal rearrangement. Here, we present seven cases with apparent isolated copy number loss, five of which showed unexpected complexity. **Methods:** Seven cases were investigated by CMA due to intellectual disability and/or dysmorphic features. Isolated deletions ranging in size from ~0.6 to ~21 Mb were found and referred for further characterization of the underlying chromosomal rearrangement. To elucidate the cases, fluorescence in situ hybridization was performed using locus-specific, whole and partial chromosome painting and/or multicolor banding. **Results:** Among the seven selected cases, there were five with unexpected complexity. Isolated deletions were actually evidence of chromoanasynthesis, ring chromosome formation, unbalanced translocation, or unbalanced insertion. **Conclusions:** These results clearly underscore that it seems reasonable to examine every case with a copy number variant—even if it appears to be “only” a simple partial deletion—using banding and/or molecular cytogenetic testing in order to make a qualified assessment of the situation and, on this basis, ensure sound genetic counseling.

## 1. Introduction

Human genetic diagnostics is a rapidly developing field. As summarized elsewhere [1], it began in 1956 with the determination of the modal chromosome number in humans as 46, continued with the introduction of banding cytogenetics and, in parallel, with the development of classical molecular genetic methods in the 1970s, such as the cloning of human DNA in vectors, polymerase chain reaction, Sanger sequencing, microsatellite analysis, and many others. Typical for the field of human genetics is that there are always new waves of current developments and usually one or two diagnostic approaches that are currently popular. In the 1990s, there was molecular cytogenetics (=fluorescence in situ hybridization; FISH), which was expected to replace band cytogenetics soon; in the 2000s, the same was true for chromosome microarray (CMA), which was expected to replace band and molecular cytogenetics within a few years. Today, or more precisely since the advent of next-generation sequencing (NGS) in this field, it is suggested that the latter approach should replace all of the above-mentioned approaches—especially since it can now also be combined with long-range sequencing = third-generation sequencing. The newest technique of optical genomic mapping (OGM), also being able to do a CMA analyses [2], is advertised in parts the same way [3]. Nevertheless, every laboratory geneticist knows that each individual approach has its advantages and shortcomings and that there is still (and most likely never will be) a single approach that can solve every diagnostic case [1].

The advantages of CMA over (molecular) cytogenetics lie in its higher resolution, which provides a genome-wide overview of euchromatic copy number variations (CNVs) [4]. CMA based on single-nucleotide polymorphisms (SNPs) can also potentially identify isodisomic segments [5]. However, CMA is blind to CNVs that are present in low mosaic form, as well as to heterochromatic CNVs; it may not even cover the entire euchromatic genome, and one must be able to distinguish meaningful CNVs from meaningless ones. Furthermore, balanced chromosomal aberrations cannot be detected, and compared to NGS, aberrations below several kilobase pairs are also not accessible [6].

In light of all these points, it is actually incomprehensible that there is currently serious discussion about completely avoiding and replacing banding and molecular cytogenetics and instead resolving cases solely through CMA and/or NGS [1]. However, the heads of diagnostic companies and institutions are certainly more easily influenced by statements in advertisements from CMA providers, as shown in Table 1 (where 8/11 statements are at least questionable), than by the more honest statements of the laboratory quality assurance committees [7]. The latter already stated the following in 2007: CMA analyses can be used as a supplement to conventional cytogenetics to further clarify chromosome abnormalities detected by G-band analysis, thereby avoiding the need for multiple FISH assays. Furthermore, it was specified that the expertise required to perform and interpret microarray data does not differ significantly from the skills and experience required for the interpretation of karyotypes and FISH, which clinical cytogeneticists already possess. Furthermore, CMA is not intended as a replacement, but rather to improve the patient care already provided by clinical cytogenetic laboratories [7]. While Miller et al. [8] stated in 2010 that “performing CMA and G-banded karyotyping on every patient substantially increases the total cost of genetic testing”, this does not neglect the necessity of testing aberrant CMA results on a chromosomal level.

Here, we present seven cases with CNVs, exclusively copy number losses, which were detected by CMA and further clarified by FISH.

## 2. Materials and Methods

### 2.1. Patients

Seven cases, each with an isolated deletion detected by CMA, were included here. All cases were investigated due to intellectual disability and/or dysmorphic features. The CMA results were provided by the physicians and their teams—see Table 2. With the exception of case 4, which has already been published [14], all cases have not been reported previously. In all cases included, it was not possible to examine the parents.

### 2.2. Molecular Cytogenetics

Fluorescence in situ hybridization (FISH) was performed with case-specific probes or probe sets. In cases 2, 3, and 7, chromosome-specific multicolor banding (MCB) probe sets for chromosomes 13, 18, and 8 were used, as previously reported [15]. In case 1, the locus-specific bacterial artificial chromosome probe RP11-36B6 in 7q32.2 was used, which was obtained from BACPAC Chori (Emeryville, CA, USA) [16] and labeled as previously described [15]. In cases 1 and 6, whole chromosome probes (wcps) obtained by microdissection were used for #7 and #13 [17], and in cases 5 and 6, a partial chromosome probe was used for all acrocentric p arms [18]. In addition, commercially available subtelomeric probes specific for 18pter, 18qter, 4pter, 4qter, 15qter, and 13qter (Abbott/Vysis, Wiesbaden, Germany) helped characterize the derivative chromosomes in cases 3 to 6. Finally, in cases 3 and 5, centromeric probes (also from Abbott/Vysis, Wiesbaden, Germany) were hybridized for chromosomes 18 and 15. All probes were hybridized according to the manufacturer’s instructions and standard procedures [15]. 4′,6-diamidino-2-phenylindole (DAPI) was used as a counterstain, which allows the chromosomes to be visualized in a G-banding-like pattern known as inverted DAPI banding.

For each case and sample set, 10–20 metaphases were analyzed using a Zeiss Axioplan microscope (Carl Zeiss, Jena, Germany) with an image analysis system (MetaSystems, Altlussheim, Germany).

## 3. Results

**Figure 1:**
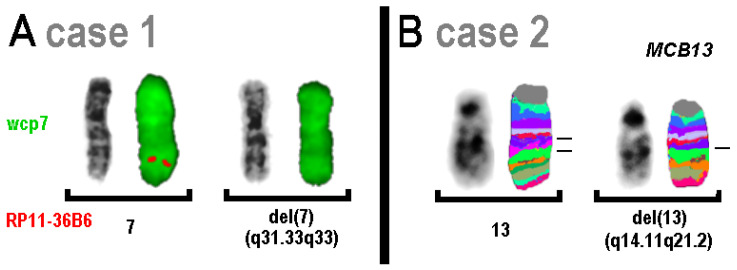
FISH results for cases 1 and 2; normal and derivative chromosomes are shown as inverted DAPI and FISH results. (**A**) The probe RP11-36B6 (red) from the deleted region gave no signal on the derivative chromosome 7; wcp7 (green) was used as the control probe. (**B**) Results of chromosome 13 specific MCB-probe set showed the deletion of 13q14.11 to 13q21.2.

CMA revealed a loss of genetic material in all seven cases examined (Table 2). In cases 1 and 2, the underlying mechanism was an isolated simple deletion of the affected region, which led to a shortening of the corresponding chromosomes. In Figure 1A, the deletion of ~11 Mb in chromosome 7 is barely visible in the cytogenetic banding pattern; however, the deletion could be clearly visualized by FISH using a locus-specific probe approximately in the middle of the affected region. However, the deletion of ~18 Mb on chromosome 13 in case 2 was clearly present in inverted DAPI band and MCB patterns.

**Figure 2:**
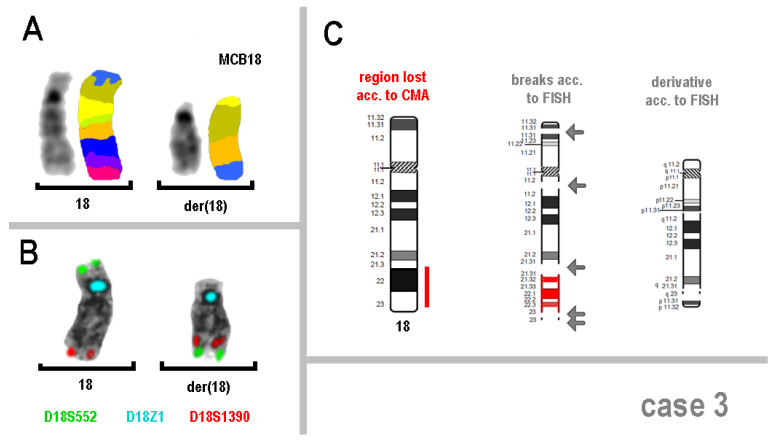
(**A**) Multicolor banding (MCB) using a probe set for chromosome 18 and locus-specific probes (**B**) resolved the complex nature of the rearrangement in chromosome 18 involving (at least) five breakpoints and the deletion of the region 18q21.32 to 18q23. In (**C)** the derivative chromosome 18 is shown schematically; arrows show the molecular cytogenetically defined breakpoints.

In case 3, however, an apparently simple deletion of ~21.1 Mb was the result of a highly complex rearrangement with at least five breakpoints. In Figure 2A, MCB18 confirms the loss of 18q21.32 to 18q23 and shows a completely rearranged band pattern compared to the normal sister chromosome 18. Figure 2B shows the colocalization of both subtelomeric probes in the long arm of the derived chromosome 18. Figure 2C shows a schematic representation of the rearrangement der(18)(:q11.2->p13.31::q11.2->q21.32::q23->q23:p13.31->pter) detected.

**Figure 3:**
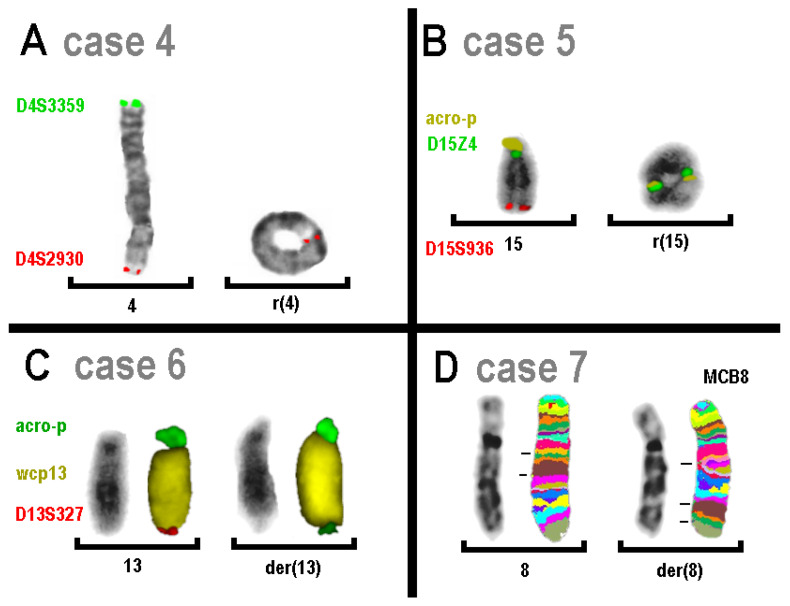
Results for cases 4 (**A**) and 5 (**B**) are shown as overlays of inverted DAPI banding and FISH signals; for cases 6 (**C**) and 7 (**D**), inverted DAPI banding and FISH results are shown side by side. (**A**) The ring chromosome 4 has a terminal deletion in 4p. (**B**) The ring chromosome 15 has terminal deletions in the short and long arm and forms a double ring in 2/10 cells. (**C**) FISH revealed a terminal deletion in the derivative chromosome 13, which carriers at its end a part of an acrocentric short arm; this part has been shown to be derived from chromosome 15p, as it was positive for D15Z1. (**D**) MCB8 could reveal an inverted insertion ins(8)(q24.3q21.13q12.1); however, the deletion was too small to be visualized by this probe set.

In case 4, CMA showed terminal deletion in the short arm of chromosome 4. This result could be confirmed in molecular cytogenetic analysis. However, unexpectedly, no terminal deletion but a ring chromosome was the underlying mechanism in this case (Figure 3A).

A similar finding was observed in case 5, which showed a ring chromosome 15 that only exhibited a terminal deletion in its long arm in the CMA. The deletion from 15pter to 15p11.2 was not detectable in the CMA, nor was the presence of a double ring chromosome 15 with low mosaicism, as shown in Figure 3B.

Case 6 showed a small terminal deletion in 13q. FISH analysis revealed the addition of p13 material from an unidentifiable acrocentric short arm at the end of the derivative chromosome 13. In case 7 (Figure 3D), a small interstitial deletion in 8q12.1 (Table 2) suggested an unbalanced insertion in chromosome 8. The region 8q12.1 to 8q21.13 was inserted in an inverted orientation into sub-band 8q24.3. Hereby, a region of ~0.6 Mb was lost in 8q12.1.

## 4. Discussion

CMA can detect gains or losses of genetic material. In a clinical case involving material loss, an isolated deletion is suspected almost immediately. In contrast, when copy number gain is detected, experienced clinicians usually request clarification of the underlying principle for CNV, as they know that “duplication” can have many different chromosomal causes. Copy number gain may be due to direct or indirect “in situ” duplication [19,20], unbalanced insertion [21] or translocation [22], the presence of a small supernumerary marker chromosome (sSMC) [23], or the result of a chromotriptic/chromoanasyntheic event [24].

This study clearly shows that chromosome deletions detected by CMA are also a reason to check the chromosome constitution of the patient and possibly also of their parents. In addition to isolated “in situ” deletions, as in cases 1 and 2, the same mechanisms as those for duplications mentioned in the previous paragraph must also be considered. In case 7, an unbalanced insertion was detected; a PubMed search yielded eight publications reporting cases with deletions caused by the same mechanism [25]. An unbalanced translocation was the underlying mechanism of the deletion in case 6; 238 articles in PubMed deal with this type of aberration [26]. In case 3, a chromosomal fusion event was identified as the cause of a deletion on chromosome 18; PubMed contained four other similar cases [27]. In the present study, there was no example of copy number loss associated with sSMC. However, there are reports of sSMCs formed by the McClintock mechanism (McCl-sSMC). These can originate from any chromosomal region and may be the cause of a mosaic deletion in a de novo McCl-sSMC carrier or a deletion in all cells of the offspring of a McCl-sSMC carrier [28,29].

The remaining cases 4 and 5 were ring chromosomes with material loss that was only present or detectable at one end of the derived chromosome. For ring chromosomes, CNV results are reported in all variants: no terminal deletions and/or one terminal deletion and/or two terminal deletions and/or (sub-)terminal duplications [30]. In acrocentric ring chromosomes (as in case 5), a terminal deletion of a short arm cannot be detected by CMA [31]. In addition, a ring duplication may be overlooked (see Figure 3B).

In conclusion, the present compilation of seven cases with deletions according to CMA underscores the need for all CMA results to be verified using a second approach. It should be noted that CMA does not cover 10% of the human genome, namely repetitive DNA and small mosaics. Both can be easily investigated using cytogenetics.

## Figures and Tables

**Table 1:** Actual statements of CMA providers on abilities of their tests.

Source/Company (Alphabetically)	Statement (Cited as Verbatim Quote)	Summary as Statements for CMA	Classification of Statements
3 billion [9]	Microarray is particularly useful for diagnosing conditions related to large chromosomal abnormalities, such as deletions, duplications, and unbalanced rearrangements. It remains a cost-effective and fast option for identifying known CNVs and SNPs in the genome.	CMA can detect chromosomal aberrations accurately CMA is more cost efficient than other approaches CMA is covering whole genome	No: CMA can detect CNVs with high accuracy, but not the nature of the chromosomal aberration No: Specialists are needed as in (molecular) cytogenetics for experiments and interpretation; machines and consumables are more expensive than in banding cytogenetics and FISH No: CMA can only cover parts of genome depending on the applied probes on the array; heterochromatic parts, euchromatic parts near heterochromatic ones and telomere-near parts are underrepresented ^1^
Agilent [10]	Testing for diseases that are genetic in origin, caused by aberrations associated with developmental delay, intellectual disability, and congenital anomalies, can vastly impact quality of life. Whether you are looking for single gene or chromosomal anomalies, chromosomal microarrays and NGS application solutions and streamlined workflows enable accurate results. Make risk-informed decisions with confidence and take your clinical research further.	Chromosomal aberrations can be picked up accurately by CMA Clinical research based on CMA provide reliable risk-informed results	No: CNVs can be picked up by CMA; not the (type of) rearrangement No: Without (molecular) cytogenetics no reliable risk-informed results can be established ^1^
Dr Lalpathlabs [11]	Most copy number variations can be interpreted based on gene content, size, inheritance, databases.	In most cases CMA is enough to solve a clinical case	No: in most cases CMA result needs to be checked by (molecular) cytogenetics ^1^
Illumina [12]	SNP arrays for cytogenetics research The identification of structural chromosomal aberrations can provide insight into causative relationships with complex phenotypes. Chromosomal microarrays leverage the investigative power of SNP genotypes to detect imbalances in copy number and allelic homozygosity, which are commonly associated with genetic constitutional disorders. Chromosomal microarrays can detect variations that may be missed by other technologies.	Cytogenetic research can be based on CMA Disease causing structural chromosomal aberrations can be identified by CMA; sometimes only by CMA CMA is superior to other approaches in detection of CNVs	No: Cytogenetic research can be based on (molecular) cytogenetics and CMA Yes: if “chromosomal aberrations” is replaced by “CNVs” Yes: concerning resolution level between (molecular) cytogenetics and NGS ^1^
PerkinElmer [13]	Cytogenomic microarrays offer a simple, reliable method for assessing chromosomal aberrations and their biological relevance at a higher resolution.	CMA is simple to do and interpret CMA has a high resolution CMA can detect chromosomal aberrations accurately CMA is more cost efficient than other approaches CMA is covering whole genome	No: Clinical Laboratory Geneticists are needed and inclusion of databases Yes: concerning resolution level between (molecular) cytogenetics and NGS No: CMA can detect CNVs with high accuracy, but not the nature of the chromosomal aberration No: Specialists are needed as in (molecular) cytogenetics for experiments and interpretation; machines and consumables are more expensive than in banding cytogenetics and FISH No: CMA can only cover parts of genome covered by the applied probes on the array; heterochromatic parts, euchromatic parts near heterochromatic ones and telomere-near parts are underrepresented ^1^

^1^ Not mentioned by any of the providers: mosaic problem.

**Table 2:** Cases included in this study.

	Results of	
Case	CMA	(Molecular) Cytogenetics
1	arr[GRCh37] 7q31.33q33(124,810,332_135,770,066)x1	46,XX.ish del(7)(RP11-36B6-) (RP11-36B6: 130,474,967-130,476,483)
2	arr[GRCh37] 13q14.11q21.2(43,067,728_61,154,642)x1	46,XY,del(13)(q14.11q21.2)
3	arr[GRCh37] 18q21.32q23(56,390,211_77,541,179)x1	46,XY,der(18)(:q11.2->p13.31::q11.2->q21.32::q23->q23:p13.31->pter)
4	arr[GRCh37] 4p16.3(98,378_997,434)x1	46,XY,r(4).ish r(4)(D4S3359-,D4S2930+) (D4S3359: position not available) (D4S2930: 189,996,850-190,197,190)
5	arr[GRCh37] 15q26.1q26.3(91,659,385_102,531,389)x1	46,XX,r(15)(p11.2q26.1)
6	arr[GRCh37] 13q34(114,052,627_115,169,824)x1	46,XY,der(13)(13pter->13q34::15p12->15pter)
7	arr[GRCh37] 8q12.1(60,206,035_60,779,958)x1	46,XX,der(8); acc. to FISH der(8) = ins(8)(q24.3q21.13q12.1)

## Data Availability

All data is included in the paper.

## Abbreviations

The following abbreviations are used in this manuscript:

CMA	chromosome microarray
CNVs	copy number variations
DAPI	4′,6-diamidino-2-phenylindole
FISH	fluorescence in situ hybridization
MCB	multicolor banding
NGS	next-generation sequencing
OGM	optical genomic mapping
pcp	partial chromosome paint
SNPs	single-nucleotide polymorphisms
sSMC	small supernumerary marker chromosome
wcp	whole chromosome paint

## References

[1] Wang Y., Liehr T. (2025). The need for a concert of cytogenomic methods in chromosomic research and diagnostics. Genes.

[2] Dharmadhikari A.V., Markowitz A.L., Han J., Estrine D.B., Xu D., Ma K., Fong C., Fernandez B.A., Deardorff M.A., Schmidt R.J. (2025). Optical genome mapping improves clinical interpretation of constitutional copy number gains and reduces their VUS burden. Genet. Med..

[3] Bionano. https://ir.bionano.com/news-releases/news-release-details/international-consortium-optical-genome-mapping-publishes-expert#:~:text=The%20consensus%20from%20a%20global,tests%20based%20on%20traditional%20methods.

[4] Coughlin C.R., Scharer G.H., Shaikh T.H. (2012). Clinical impact of copy number variation analysis using high-resolution microarray technologies: Advantages, limitations and concerns. Genome Med..

[5] Cheung S.W., Bi W. (2018). Novel applications of array comparative genomic hybridization in molecular diagnostics. Expert Rev. Mol. Diagn..

[6] Liehr T. (2021). Molecular cytogenetics in the era of chromosomics and cytogenomic approaches. Front. Genet..

[7] Shaffer L.G., Beaudet A.L., Brothman A.R., Hirsch B., Levy B., Martin C.L., Mascarello J.T., Rao K.W., Working Group of the Laboratory Quality Assurance Committee of the American College of Medical Genetics (2007). Microarray analysis for constitutional cytogenetic abnormalities. Genet. Med..

[8] Miller D.T., Adam M.P., Aradhya S., Biesecker L.G., Brothman A.R., Carter N.P., Church D.M., Crolla J.A., Eichler E.E., Epstein C.J. (2010). Consensus statement: Chromosomal microarray is a first-tier clinical diagnostic test for individuals with developmental disabilities or congenital anomalies. Am. J. Hum. Genet..

[9] 3Billion. https://3billion.io/blog/microarray-vs-wes.

[10] Agilent. https://www.agilent.com/en/solutions/inherited-disease/postnatal?&utm_source=bing&utm_medium=cpc&utm_campaign=B_PS_NBr_HRG_EMEA_B_E_P&utm_term=chromosomal%20microarray&utm_content=Postnatal_P&gclid=09c7c545d1e913fc8014a7d3d94445fe&gclsrc=3p.ds.

[11] Dr Lalpathlabs. https://www.lalpathlabs.com/images/our-department/cytogenetics/knowledge-centre/Microarrays.pdf.

[12] Illumina. https://www.illumina.com/areas-of-interest/genetic-disease/rare-disease-genomics/cma-constitutional-cytogenetics.html.

[13] PerkinElmer. https://perkinelmer-appliedgenomics.com/home/products/molecular-cytogenetics/cytogenetic-microarrays/.

[14] Soysal Y., Balci S., Hekimler K., Liehr T., Ewers E., Schoumans J., Bui T.H., Içduygu F.M., Kosyakova N., Imirzalioğlu N. (2009). Characterization of double ring chromosome 4 mosaicism associated with bilateral hip dislocation, cortical dysgenesis, and epilepsy. Am. J. Med. Genet. A.

[15] Weise A., Mrasek K., Fickelscher I., Claussen U., Cheung S.W., Cai W.W., Liehr T., Kosyakova N. (2008). Molecular definition of high-resolution multicolor banding probes: First within the human DNA sequence anchored FISH banding probe set. J. Histochem. Cytochem..

[16] BACPAC Resources Center (BPRC). https://bacpacresources.org.

[17] Mrasek K., Heller A., Rubtsov N., Trifonov V., Starke H., Claussen U., Liehr T. (2003). Detailed Hylobates lar karyotype defined by 25-color FISH and multicolor banding. Int. J. Mol. Med..

[18] Liehr T., Claussen U. (2002). Current developments in human molecular cytogenetic techniques. Curr. Mol. Med..

[19] De Gregori M., Pramparo T., Memo L., Gimelli G., Messa J., Rocchi M., Patricelli M.G., Ciccone R., Giorda R., Zuffardi O. (2005). Direct duplication 12p11.21-p13.31 mediated by segmental duplications: A new recurrent rearrangement?. Hum. Genet..

[20] Eussen B.H., van de Laar I., Douben H., van Kempen L., Hochstenbach R., De Man S.A., Van Opstal D., de Klein A., Poddighe P.J. (2007). A familial inverted duplication 2q33-q34 identified and delineated by multiple cytogenetic techniques. Eur. J. Med. Genet..

[21] Neill N.J., Ballif B.C., Lamb A.N., Parikh S., Ravnan J.B., Schultz R.A., Torchia B.S., Rosenfeld J.A., Shaffer L.G. (2011). Recurrence, submicroscopic complexity, and potential clinical relevance of copy gains detected by array CGH that are shown to be unbalanced insertions by FISH. Genome Res..

[22] Debost-Legrand A., Capri Y., Gouas L., Pebrel-Richard C., Veronese L., Tchirkov A., Haoud K., Boespflug-Tanguy O., Goumy C., Vago P. (2011). De novo unbalanced translocation 2;4 characterized by metaphase CGH and array CGH in a child with mental retardation and dysmorphic features. Pathol. Biol..

[23] González-Rodríguez M.T.A., Brukman-Jiménez S.A., Cuero-Quezada I., Corona-Rivera J.R., Corona-Rivera A., Serafín-Saucedo G., Aguirre-Salas L.M., Bobadilla-Morales L. (2023). Identification of a small supernumerary marker chromosome in a Turner syndrome patient with karyotype mos 46,X,+mar/45,X. Genes.

[24] Imaizumi T., Yamamoto-Shimojima K., Yanagishita T., Ondo Y., Nishi E., Okamoto N., Yamamoto T. (2020). Complex chromosomal rearrangements of human chromosome 21 in a patient manifesting clinical features partially overlapped with that of Down syndrome. Hum. Genet..

[25] PubMed Searching for Array CGH Unbalanced Insertion Deletion. https://pubmed.ncbi.nlm.nih.gov/?term=array%20cgh%20unbalanced%20insertion%20deletion&sort=date.

[26] PubMed Searching for Array CGH Unbalanced Translocation Deletion. https://pubmed.ncbi.nlm.nih.gov/?term=array+cgh+unbalanced+translocation+deletion&sort=date.

[27] PubMed Searching for Array CGH Chromoanasynthesis Deletion. https://pubmed.ncbi.nlm.nih.gov/?term=array+cgh+chromoanasynthesis+deletion&sort=date.

[28] Quinonez S.C., Gelehrter T.D., Uhlmann W.R. (2017). A Marfan syndrome-like phenotype caused by a neocentromeric supernumerary ring chromosome 15. Am. J. Med. Genet. A.

[29] Liehr T. (2023). Small Supernumerary Marker Chromosomes, Basics.

[30] Conlin L.K., Kramer W., Hutchinson A.L., Li X., Riethman H., Hakonarson H., Mulley J.C., Scheffer I.E., Berkovic S.F., Hosain S.A. (2011). Molecular analysis of ring chromosome 20 syndrome reveals two distinct groups of patients. J. Med. Genet..

[31] Knijnenburg J., van Haeringen A., Hansson K.B., Lankester A., Smit M.J., Belfroid R.D., Bakker E., Rosenberg C., Tanke H.J., Szuhai K. (2007). Ring chromosome formation as a novel escape mechanism in patients with inverted duplication and terminal deletion. Eur. J. Hum. Genet..

